# Clinical and hematological analysis of testicular torsion in children

**DOI:** 10.3389/fped.2024.1399349

**Published:** 2024-09-20

**Authors:** Qi-Fei Deng, Chao Yang, Changkun Mao, Han Chu

**Affiliations:** The Second Department of Pediatric Urology Surgery, Anhui Provincial Children’s Hospital, Children’s Hospital of Fudan University-Anhui Campus, Hefei, Anhui, China

**Keywords:** pediatric testicular torsion, orchidopexy, multivariate logistic regression analysis, hematological parameters, hydrocele

## Abstract

**Purpose:**

Analyze the clinical manifestations, laboratory tests, and imaging data of testicular torsion to provide clinical insights for timely and accurate diagnosis and treatment of testicular torsion.

**Methods:**

A retrospective analysis was conducted on the clinical data of 67 pediatric patients suspected of testicular torsion, admitted and subjected to surgical exploration from June 2018 to June 2023. Based on whether the torsed testicle was excised during surgery, the patients were divided into orchiectomy group (40 cases) and orchidopexy group (27 cases). Combining clinical symptoms, signs, ultrasound examinations, and laboratory tests, the study aimed to summarize the influencing factors on the onset, diagnosis, and treatment of testicular torsion.

**Results:**

The clinical manifestations of all 67 pediatric patients were generally typical. Color Doppler Flow Imaging (CDFI) and surgical exploration were performed for all cases, and the results were consistent. Testicular color doppler ultrasound suggested reduced or absent blood flow, leading to surgical treatment in all cases. All patients had unilateral testicular torsion, with 46 cases (68.66%) on the left side and 21 cases (31.34%) on the right side. Intrafunicular torsion occurred in 60 cases (89.55%), while extrafunicular torsion occurred in 7 cases (10.45%). The onset distribution was as follows: 20 cases in spring, 16 cases in summer, 16 cases in autumn, and 15 cases in winter. Univariate analysis indicated significant statistical differences in age, degree of testicular torsion, duration of symptoms, NEUT, NLR, and occurrence of tunica fluid between the two groups of patients. Multivariate logistic regression analysis showed that the duration of symptoms and the occurrence of hydrocele were independent risk factors for determining testicular viability.

**Conclusion:**

Testicular torsion is more common in children and adolescents, with clinical manifestations including scrotal pain, scrotal redness and swelling, abdominal pain, nausea, and vomiting. In the early stages of testicular torsion, inflammatory markers in the blood increase, and preoperative ultrasound indicates hydrocele. This suggests that the testicle is in an early twisted state, with good viability and potential for preservation.

## Introduction

Testicular torsion is commonly encountered in pediatric scrotal emergencies and occur at various age groups. The etiology is not fully elucidated, often attributed to anomalies in the anatomical structure of the testis or spermatic cord, or excessive mobility of the testis leading to torsion (1, 2). Its clinical manifestations are diverse, with common typical symptoms of scrotal redness and swelling, accompanied by pain. However, some patients may exhibit atypical scrotal symptoms, such as abdominal pain, nausea, vomiting, and other non-specific symptoms. In clinical practice, it is prone to misdiagnosis as urinary stones, appendicitis, or intestinal inflammation. Without early identification and treatment, there is a risk of delaying the treatment and leading to potential testicular necrosis (3). Clinical suspicion of testicular torsion should prompt early surgical exploration to prevent misdiagnosis and missed diagnosis. Currently, the diagnosis of testicular torsion mainly relying on clinical symptoms, physical examination, and color Doppler ultrasound. Ultrasound plays a crucial role in the examination of testicular torsion, but it may have limitations in cases of incomplete or early torsion, with atypical presentations that could be influenced by the subjectivity of the ultrasound operator, leading to the possibility of misdiagnosis or missed diagnosis (4). This study retrospectively analyzed the clinical data and treatment outcomes of 67 pediatric patients with testicular torsion admitted to the Children's Hospital of Anhui Province from June 2018 to June 2023. It aimed to explore the predictive value of preoperative imaging data, blood parameters and clinical manifestations for the viability of the testicular torsion in children.

## Materials and methods

**Figure 1:**
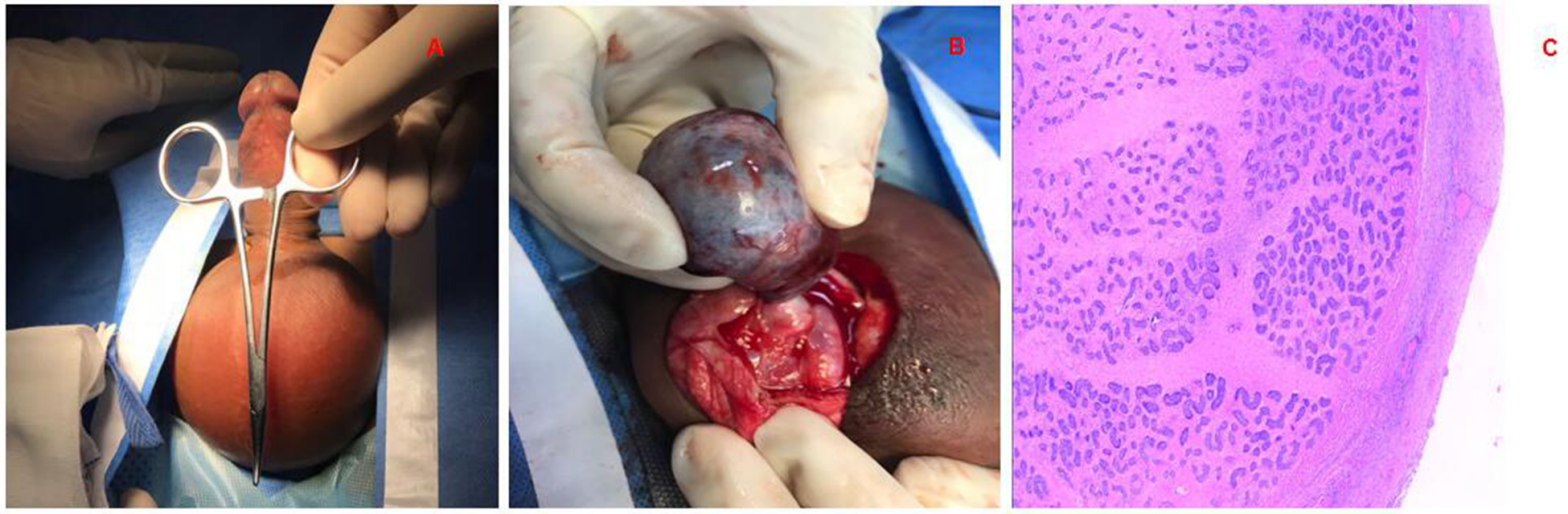
Testicular torsion. **(A)** Skin of the scrotum is red, the testicles are swollen, and the cremaster reflex is gone; **(B)** operation revealed that the testicle had become necrotic with intramural torsion. **(C)** Postoperative pathology showed hemorrhagic necrosis of the testicle.

1. General Information Selecting scrotal emergency pediatric patients who visited our hospital and underwent surgical exploration from June 2018 to June 2023, a total of 67 cases. Inclusion criteria: (1) Pediatric patients aged below 16 years; (2) Scrotal ultrasound indicating reduced testicular blood supply, unclear testicular blood supply, or absence of testicular blood supply, prompting preoperative suspicion of testicular torsion and the need for testicular exploration surgery to confirm the presence of torsion; (3) Postoperative pathological confirmation of testicular necrosis. Exclusion criteria: (1) History of scrotal surgery or concurrent inflammatory diseases; (2) Intraoperatively confirmed testicular necrosis, but family requests to retain the necrotic testicle (Figure 1). Based on whether the torsed testicle was excised during surgery, patients were divided into orchiectomy group (40 cases) and orchidopexy group (27 cases). This study was approved by the Ethics Committee of Anhui Children's Hospital (ETYY-2023101).
2. Collect clinical data of patients, including clinical symptoms (such as scrotal redness, tenderness, vomiting, onset time, etc.), preoperative scrotal ultrasound results, laboratory tests including white blood cell count (WBC), neutrophil count (NEUT), platelet count (PLT), plateletcrit (PCT), mean platelet volume (MPV), platelet distribution width (PDW), lymphocyte count (LYM), neutrophil- lymphocyte ratio (NLR), platelet-lymphocyte ratio (PLR), and intraoperative testicular torsion status.
3. Diagnosis and Treatment: All 67 pediatric underwent surgical exploration. After confirming testicular torsion intraoperatively, all torsed testicles underwent detorsion. Following detorsion, warm normal saline immersion was performed for 15 min to observe the blood supply to the detorsed testicle. The decision on whether to retain the testicle was made according to the method reported by Arda et al. (5), involving needle puncture deep into the testicular parenchyma, observing the bleeding time. The criteria were as follows: Grade I: immediate appearance of fresh red blood; Grade II: no immediate bleeding but bleeding observed within 10 min; Grade III: no bleeding even after 10 min. For patients achieving Grade I or II, testicular repositioning and fixation surgery on the affected side and fixation surgery on the unaffected side were performed. For Grade III patients, the affected side underwent testicular excision, and fixation surgery was performed on the unaffected side.
4. All patients were followed up at 6 months postoperatively, including medical history, physical examination, and scrotal ultrasound examination.
5. Statistical Analysis: Statistical analysis was performed using SPSS 26.0. Normally distributed continuous data are expressed as mean ± standard ($\bar{x}\pm s$), and non-normally distributed data are presented as median M(Q1, Q3). Count data are expressed as rates (%). Independent sample *t*-tests were employed for normally distributed data, while the Mann-Whitney U test was used for non-normally distributed data to compare between two groups. The *χ*^2^ test or Fisher's exact test was used for comparison between groups. Variables with *P* < 0.05 in univariate analysis were included in multivariate logistic regression analysis. Receiver Operating Characteristic (ROC) curve analysis was conducted, and the Area Under the Curve (AUC) was calculated. *P* < 0.05 was considered statistically significant.

## Results

All 67 patients with testicular torsion experienced unilateral testicular torsion (Figure 1). Clinical manifestations included scrotal pain in 64 cases (95.52%), scrotal redness and swelling in 61 cases (91.04%), and nausea/vomiting in 8 cases (11.94%). Preoperative ultrasound indicated reduced or absent blood supply in 66 patients, with unclear blood supply in 1 patient. Additionally, 34 patients had preoperative ultrasound indicating tunica fluid. In terms of torsion characteristics, 60 cases (89.55%) were intrafunicular torsion, and 7 cases (10.45%) were extrafunicular torsion. Left-sided torsion occurred in 46 cases, right-sided in 21 cases. Onset distribution by seasons included 20 cases in spring, 16 cases in summer, 16 cases in autumn, and 15 cases in winter.

Among the 67 patients with testicular torsion, 40 underwent testicular excision surgery, with 27 on the left side and 13 on the right side. The average age was 61.50 (2.1, 144.0) months, with 22 cases being ≤72 months, 3 cases between 72 and 120 months, and 15 cases ≥120 months; the median torsion angle was 720° (360°–720°), with 12 cases having a torsion angle of ≤360° and 28 cases having a torsion angle of ≥360°. The median time from onset to surgery was 48 (24, 120) hours, including 3 cases with torsion time ≤6 h, 4 cases with 6–24 h, and 33 cases with ≥24 h. Additionally, 27 patients underwent orchidopexy surgery, with 19 on the left side and 8 on the right side. The median age was 144.0 (84.0, 156.0) months, with 5 cases being ≤72 months, 4 cases between 72 and 120 months, and 18 cases ≥120 months; the median torsion angle was 360° (180°–720°), with 15 cases having a torsion angle of ≤360° and 12° cases having a torsion angle of ≥360°. The median time from onset to surgery was 8 (3, 24) hours, including 13 cases with torsion time ≤6 h, 7 cases with 6–24 h, and 7 cases with ≥24 h (Table 1A, B).

**Table 1A:** Baseline characteristics of patients with testicular torsion in children.

Variables	Orchidopexy (*n* = 27)	Orchiectomy (*n* = 40)	Statistic	*P*
Age (month)			*χ*^2 ^= 8.933	0.011
≤72	5	22		
72–120	4	3		
≥120	18	15		
Symptoms duration (h)			*χ*^2 ^= 22.285	<0.01
≤6	13	3^#^		
6–12	7	4		
≥24	7	33		
Torsion degree (°)			*χ*^2 ^= 4.376	0.036
≤360	15	12		
>360	12	28		

#: For these three children, intraoperative findings showed a torsion degree greater than 360 degrees. After repositioning and soaking in warm saline, and performing the Arda test, the testicles still showed no blood flow, confirming irreversible testicular necrosis, and were subsequently removed.

**Table 1B:** Baseline characteristics of patients with testicular torsion in children.

Variables	Total (*n* = 67)	Orchidopexy (*n* = 27)	Orchiectomy (*n* = 40)	Statistic	*P*
Age (month) M (Q₁, Q₃)	108.00 (34.00, 156.00)	144.00 (90.00, 156.00)	61.50 (5.08, 144.00)	*Z* = −2.98	0.003
Symptoms duration (h), M (Q₁, Q₃)	24.00 (7.00, 48.00)	8.00 (3.00, 22.00)	48.00 (24.00, 120.00)	*Z* = −4.50	<.001
Torsion degree (°) M (Q₁, Q₃)	720.00 (360.00, 720.00)	360.00 (180.00, 720.00)	720.00 (360.00,720.00)	*Z* = −2.05	0.041
Tender scrotum, *n* (%)				*χ*^2 ^= 0.73	0.393
No	3 (4.48)	0 (0.00)	3 (7.50)		
Yes	64 (95.52)	27 (100.00)	37 (92.50)		
Vomit, *n* (%)				*χ*^2 ^= 3.06	0.080
No	59 (88.06)	21 (77.78)	38 (95.00)		
Yes	8 (11.94)	6 (22.22)	2 (5.00)		
Testicular swelling, *n* (%)				*χ*^2 ^= 0.64	0.423
No	6 (8.96)	1 (3.70)	5 (12.50)		
Yes	61 (91.04)	26 (96.30)	35 (87.50)		
Hydrocele, *n* (%)				*χ*^2 ^= 13.22	<.001
No	33 (49.25)	6 (22.22)	27 (67.50)		
Yes	34 (50.75)	21 (77.78)	13 (32.50)		
Laterality, *n* (%)				*χ*^2 ^= 0.06	0.804
Left	46 (68.66)	19 (70.37)	27 (67.50)		
Right	21 (31.34)	8 (29.63)	13 (32.50)		
Torsion direction, *n* (%)				*χ*^2 ^= 4.19	0.041
Anticlockwise	35 (52.24)	10 (37.04)	25 (62.50)		
Clockwise	32 (47.76)	17 (62.96)	15 (37.50)		
Torsion position with the cavity, *n* (%)				*χ*^2 ^= 0.07	0.794
Inside	60 (89.55)	25 (92.59)	35 (87.50)		
Outside	7 (10.45)	2 (7.41)	5 (12.50)		

*Z*: Mann-Whitney test, *χ*^2^: Chi-square test; M: Median, Q₁: 1st Quartile, Q₃: 3st Quartile.

Among the 27 patients who underwent testis-sparing surgery, 2 were lost to follow-up. The remaining 25 patients underwent scrotal ultrasound re-examinations 6 months after the surgery. Among them, 20 testes survived, while 5 testes (20%) atrophied. Fisher's exact test indicated a statistically significant difference between the two groups (*P* < 0.05), as shown in Table 2.

**Table 2:** Postoperative testicular atrophy and testis survival in the testicular torsion patients in different grades.

Variables	Testicular atrophy *N* (%)	Testis survival *N* (%)	Statistic	*P*
Arda Grade分级			–	**0.039**
I级	0 (0.00)	12 (100.00)		
II级	5 (38.46)	8 (61.54)		

–: Fisher exact.

The bold value suggests *p* < 0.05, statistically significant.

Univariate analysis (Table 3) showed statistically significant differences (*P* < 0.05) between the testicular repositioning and fixation group and the testicular excision group in terms of testicular torsion angle, duration of symptoms, NEUT, NLR, and tunica fluid. No statistically significant differences (*P* > 0.05) were observed in WBC, LYM, MXD, PLT, PCT, MPV, PDW, and PLR.

**Table 3:** Hematological parameters characteristics of testicular torsion in children.

Variables	Total (*n* = 67)	Orchidopexy (*n* = 27)	Orchiectomy (*n* = 40)	Statistic	*P*
WBC (10^9^/L)	11.25 ± 3.07	11.75 ± 3.04	10.90 ± 3.08	*t* = 1.12	0.268
NEUT (10^9^/L)	7.33 ± 3.18	8.44 ± 3.34	6.59 ± 2.88	*t* = 2.42	0.019
PLT (10^9^/L)	303.76 ± 84.42	282.56 ± 66.38	318.07 ± 92.74	*t* = −1.71	0.091
PCT (%) M (Q₁, Q₃)	0.28 (0.25, 0.34)	0.27 (0.24, 0.30)	0.28 (0.26, 0.35)	*Z* = −1.16	0.244
MPV(fl) M (Q₁, Q₃)	9.70 (9.15, 10.60)	9.80 (9.40, 10.55)	9.50 (9.07, 10.60)	*Z* = −1.19	0.234
PDW(fl) M (Q₁, Q₃)	12.70 (9.90, 15.80)	12.70 (10.65, 15.85)	12.55 (9.80, 15.72)	*Z* = −0.95	0.344
LYM(10^9^/L) M (Q₁, Q₃)	2.66 (1.74, 3.60)	2.12 (1.48, 3.36)	2.84 (1.94, 3.66)	*Z* = −1.50	0.133
NLR M (Q₁, Q₃)	2.91 (1.42, 4.94)	3.59 (2.04, 6.40)	2.65 (1.36, 3.65)	*Z* = −2.24	0.025
PLR M (Q₁, Q₃)	113.64 (83.28,157.80)	128.42 (81.47, 164.31)	110.87 (84.76, 146.00)	*Z* = −0.86	0.391

WBC, white blood cell; NEUT, neutral cell; PLT, platelet; PCT, platelet accumulation; MPV, mean platelet volume; PDW, platelet distribution width; LYM, lymphocyte; NLR, neutrocyte-lymphocyte ratio; PLR, platelet-lymphocyte ratio. SD, standard deviation. *t*-test, *Z*: Mann-Whitney test SD: standard deviation, M: Median, Q₁: 1st Quartile, Q₃: 3st Quartile.

The factors with *P* < 0.05 in the univariate analysis were included in the multivariate logistic regression analysis (Table 4). The results showed that the duration of symptoms (OR = 1.05, *P* = 0.013) and tunica fluid (OR = 0.18, *P* = 0.021) were independent risk factors for testicular viability (*P* < 0.05).

**Table 4:** Univariate and multivariate analyses results.

Variables	Univariate analysis			Multivariate analysis		
	OR	(95% CI)	*P*	OR	(95% CI)	*P*
Age (month)	0.99	(0.98–0.99)	0.004	0.99	(0.97–0.99)	0.046
Symptoms.Duration (h)	1.05	(1.02–1.09)	0.003	1.05	(1.01–1.10)	0.013
Torsion degree (°)	1.01	(1.01–1.01)	0.028	1.00	(1.00–1.00)	0.796
NEUT (10^9^/L)	0.82	(0.69–0.97)	0.024	1.16	(0.82–1.63)	0.398
NLR	0.75	(0.60–0.95)	0.015	0.97	(0.71–1.32)	0.826
Hydrocele	0.14	(0.04–0.42)	<.001	0.18	(0.04–0.77)	0.021

NEUT, neutral cell; NLR, neutrocyte-lymphocyte ratio.

**Figure 2:**
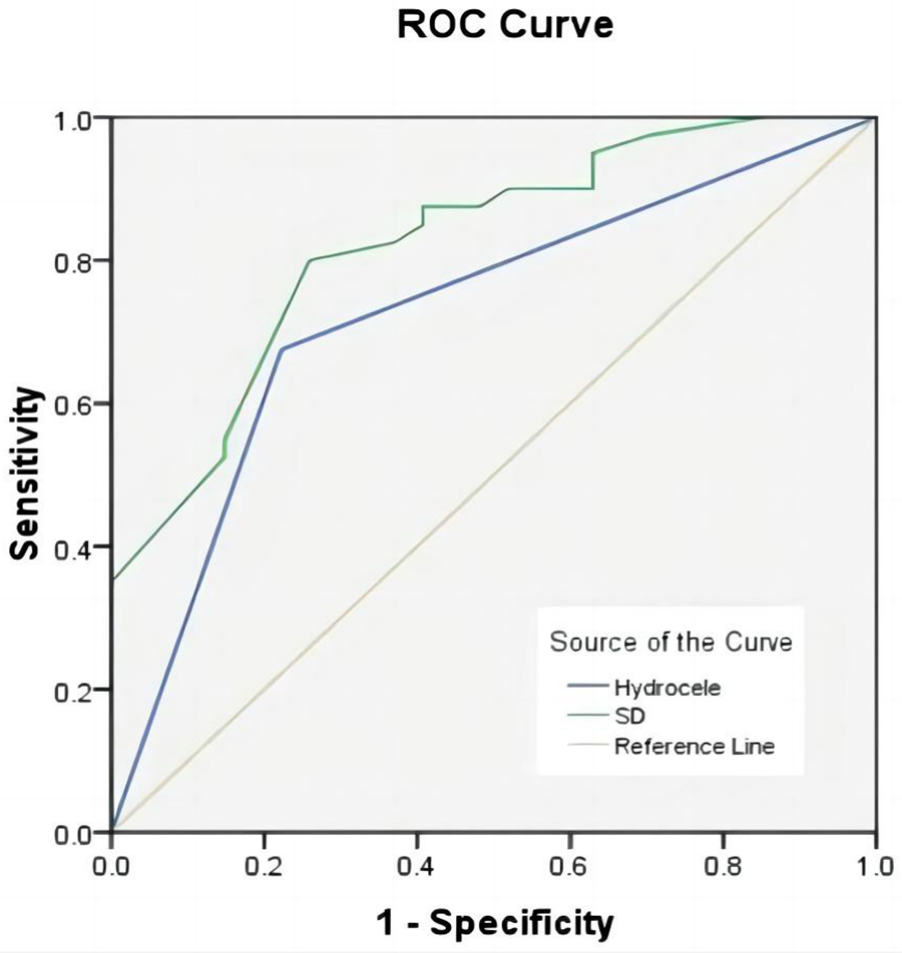
ROC curves of the hydrocele, symptom duration (SD) for predicting testicular salvage.

The ability of clinical symptoms and hematological parameters to predict testicular viability after torsion was further assessed using ROC curves. ROC analysis showed that the AUC for the duration of symptoms was 0.825 (*P* < 0.01, sensitivity 74.1%, specificity 80.0%, critical value 15 h), and the AUC for tunica fluid was 0.726 (*P* < 0.01, sensitivity 77.8%, specificity 67.5%) (Figure 2).

## Discussion

Pediatric testicular torsion, also known as spermatic cord torsion, often occurs during vigorous physical activity, changes in body position, or in situations involving abrupt temperature changes or violent injuries. The cremaster muscle, attached to the spermatic cord, undergoes sudden strong contractions, causing torsion of the testicle (6). Clinically, testicular torsion is classified into intravaginal and extravaginal types based on the location of torsion. Intravaginal torsion is more common, accounting for over 90%, and is characterized by an excessively long or absent gubernaculum, lacking appropriate fixation structures. Extravaginal torsion is often seen in newborns or infants and is attributed to incomplete attachment of the testicular cord to the scrotal wall. The spermatic cord and the tunica sac rotate together, with the torsion occurring higher than intravaginal torsion, often near the external inguinal ring (7). The present study found that intravaginal torsion occurred in 60 cases (89.55%), while extravaginal torsion occurred in 7 cases (10.45%). Pediatric testicular torsion has a bimodal age distribution, primarily occurring during adolescence and shortly after birth. According to literature, the incidence is approximately 0.004% in patients under 18 years of age (8). In this study, patients aged below 6 and above 10 accounted for 60 cases (89.55%), consistent with previous research. Testicular torsion typically affects one side and is more common on the left, with the exact reason unknown, possibly related to the longer left spermatic cord (9). In our study, 46 cases (68.66%) were on the left side, and 21 cases (31.34%) were on the right side.

Patients with testicular torsion typically present with noticeable clinical symptoms. Approximately 70%–90% experience scrotal pain, 60%–75% develop testicular or scrotal swelling, 7%–28% may have abdominal pain, and 5%–43% may exhibit nausea or vomiting (10). In this study, clinical manifestations included scrotal pain in 64 cases (95.52%), scrotal redness and swelling in 61 cases (91.04%), and nausea/vomiting in 8 cases (11.94%). The testicular pain in most patients is sudden and can radiate to the groin or lower abdomen, accompanied by nausea, vomiting, and fever. Therefore, in cases of acute onset, abdominal pain, or vomiting in children and adolescents, the possibility of testicular torsion should be considered. A thorough examination can help avoid misdiagnosis and missed diagnosis (11). For scrotal pain, it is crucial to promptly understand the cause, identify testicular torsion, and initiate treatment to prevent testicular dysfunction. It is noteworthy that recurrent severe pain or spontaneous resolution may suggest intermittent testicular torsion or self-repositioning after mild torsion. Therefore, in shy or embarrassed adolescents, inquiring about medical history and obtaining clues from parents is crucial (12).

Patients suspected of testicular torsion require a detailed physical examination, including examination of the genitalia, groin, and abdomen. Testicular examination can be performed in a supine or standing position. Compared to the relaxed and soft contralateral testicle, the affected testicle will exhibit tension towards the abdominal cavity, becoming harder and more tender. The most crucial auxiliary examination is scrotal color Doppler ultrasonography. The sensitivity of ultrasound diagnosis for testicular torsion ranges from 63.6% to 100%, with specificity from 97% to 100% (13). In this study, 63 cases (94.03%) showed no blood flow in the testicle, while 4 cases (5.97%) indicated decreased blood flow or unclear blood flow signals. The overall positive rate of color Doppler ultrasonography was relatively high, which is also related to the expertise and experience of the ultrasound examiner. Simultaneously, our research found that the incidence of hydrocele in the testicular fixation group was significantly higher than in the testicular excision group, with statistical significance. However, ultrasound results can only serve as a reference. In the early stages or intermittent torsion stages of testicular torsion, arterial blood flow may lead to false-negative results. In a multicenter study of 208 cases of testicular torsion, 24% of patients had normal testicular blood flow in the early stages of torsion (14). Therefore, in cases where testicular torsion is suspected, surgical exploration is necessary. Our article finds that both the duration of ischemia and the degree of torsion are important factors in determining whether the testicle can be preserved. In orchidopexy group, with 15 cases (55.56%) having a torsion degree of ≤360° and 12 cases (44.44%) having a torsion degree of >360°; In orchiectomy group, with 12 cases (30%) having a torsion angle of ≤360° and 28 cases (70%) having a torsion angle of >360°. The statistical results also indicate a significant difference between the two groups (*P* < 0.05), demonstrating that the degree of testicular torsion can affect the rate of testicular removal. Our study also found that the proportion of cases with onset time exceeding 24 h in the testicular excision group was 82.5%, while in the testicular fixation group, it was only 25.93%. Therefore, early emergency surgical exploration is the optimal treatment. We do not recommend manual reduction as it may delay treatment and could lead to torsion recurrence after reduction.

According to current research reports, even after detorsion of testicular torsion, there is still a 12%–68% chance of eventual testicular atrophy (15). In our study, among the 25 patients who were followed up after surgery, 5 cases (20%) developed testicular atrophy. According to the Arda classification, testicular recovery was good in Grade I cases. However, 5 cases of testicular atrophy occurred in Grade II, all of which were in the group where the onset was more than 24 h. This suggests that the damage caused by testicular torsion is irreversible, and the longer the ischemic time, the greater the testicular damage. In this group of patients, there were 2 cases where the onset time exceeded 24 h, and testis preservation surgery was performed. During follow-up, blood flow was observed in the affected testis. For these two cases, In these two cases, it is possible that there was still a small amount of blood flow to the testicles, which reduced ischemic damage. Another possibility is the presence of intermittent testicular torsion, where repeated episodes of torsion may spontaneously resolve, thus prolonging the duration of symptoms while actually shortening the time of testicular ischemia (16).

Multiple studies have reported the reference value of hematological parameters in the grading of inflammatory diseases, diagnosis of cardiovascular diseases, and differentiation of benign and malignant tumors (17–19). However, there is limited research on the diagnostic value of hematological parameters in testicular torsion. Jang et al. (20) reported preoperative NLR can serve as a predictive indicator of testicular activity in patients with testicular torsion undergoing fixation and those undergoing testicular excision within the torsion time window of 3 to 12 h. Merder's study found a difference in preoperative monocyte count in children with testicular torsion between the fixation and excision groups (21). Similarly, research has shown that MPV can not only serve as a predictive indicator for preoperative assessment of testicular vitality but also predict postoperative atrophy of the repositioned testicle (22). In our study, a single-factor analysis of the whole blood parameters in the testicular fixation group and the testicular excision group found statistically significant differences in age (*P* = 0.0025), duration of illness (*P* < 0.001), degree of testicular torsion (*P* = 0.0393), NEUT (*P* = 0.0185), and NLR (0.0248) between the two groups. Subsequently, the indicators with *P* < 0.05 in the single-factor analysis were included in the multifactor analysis. The duration of symptoms and the absence of hydrocele in preoperative ultrasound were independent risk factors for testicular excision. There was no statistical significance between NLR and NEUT in the two groups, which is inconsistent with previous studies. We consider this discrepancy may be related to the relatively small number of cases and the wide age range of children included in this study.

Blood parameters such as NEUT, NLR, PLR, and MPV play a crucial role in the inflammatory progression and have been increasingly used in the diagnosis of acute and chronic inflammation. Several studies have found that these markers can be applied to the diagnosis and prognosis of cancer (23). A study comparing patients with testicular torsion to a control group found a significant increase in PLR and NLR (24). In our study, we observed a statistically significant increase in inflammatory markers NLR and NEUT in the orchidopexyn group compared to the orchiectom group (*p* < 0.05). Additionally, we found that the occurrence of hydrocele in the testicular fixation group and the testicular excision group had statistical significance (*p* < 0.05). We consider that when testicular torsion occurs in the early stages, there is an elevation of inflammatory markers in the blood, and preoperative ultrasound suggests hydrocele. This to some extent indicates that the testicle is in an early torsion state with good activity and has preservable value. During surgery, consideration can be given to performing testicular repositioning and fixation.

The exploration of NLR in this study provides a feasible direction for addressing this issue. Routine blood tests serve as an objective, economical, and widely applicable diagnostic tool, especially in emergency situations. They can provide more effective information for preoperative evaluation in such cases. However, this study has some limitations: Firstly, it is a retrospective study, and there is a need to expand the sample size and conduct further prospective research to confirm the predictive role of NLR in testicular activity and atrophy. Secondly, testicular torsion is considered an emergency surgery, and although the physical examination upon admission and preoperative ultrasound were performed by experienced physicians, the involvement of different doctors may introduce some variability. Lastly, the wide age range of pediatric patients in the study could potentially lead to biased results.

## Conclusion

Testicular torsion is more common in children and adolescents, presenting with symptoms such as scrotal pain, scrotal swelling, abdominal pain, and nausea/vomiting. Scrotal Doppler ultrasound is effective in observing testicular blood flow, and surgery is the most accurate treatment method. In the early stages of testicular torsion, there is an elevation in inflammatory markers in the blood, and preoperative ultrasound may indicate scrotal fluid accumulation. These findings suggest that the testicle is in an early twisted state, with good viability, and holds potential for preservation.

## Data Availability

The raw data supporting the conclusions of this article will be made available by the authors, without undue reservation.
